# Supplementary material for: PPM1G forms a PPP‐type phosphatase holoenzyme with B56δ that maintains adherens junction integrity
Source: EMBO Rep. 2019 Aug 21;20(10):e46965. doi: 10.15252/embr.201846965 (PMC6776900; doi:10.15252/embr.201846965)

Appendix

PPM1G forms a PPP-type phosphatase holoenzyme with B56δ that maintains adherens junction integrity

Parveen Kumar[1, 2], Prajakta Tathe[1, 2], Neelam Chaudhary[1] & Subbareddy Maddika[1]*

[1]Laboratory of Cell Death & Cell Survival, Centre for DNA Fingerprinting and Diagnostics (CDFD), Uppal, Hyderabad 500039, INDIA

[2]Graduate studies, Manipal Academy of Higher Education, Manipal 576104, INDIA

*To whom correspondence should be addressed.

Dr. Subbareddy Maddika

Tel: +91-40-2721 6168

Email: msreddy@cdfd.org.in

List of Appendix Figure Legends and Figures:

Appendix Figure S1 legend

Appendix Figure S2 legend

Appendix Figure S3 legend

Appendix Figure S4 legend

Appendix Figure S1

Appendix Figure S2

Appendix Figure S3

Appendix Figure S4

Appendix Figure S1: Multiple sequence alignment of B56 family members generated using CLUSTAL-Omega is shown.

Appendix Figure S2: **(A)** Amount of PPM1G in cytoplasmic and nuclear fractions was calculated by quantifying the data by using ImageJ from figure 2D, (n=2 independent experiments). **(B)** Quantification of data from figure 2E, derived by using ImageJ is shown (n=2 independent experiments).

Appendix Figure S3: **(A-K)** Quantification of data from **(A)** figure 3B, **(B)** figure 3E, **(C)** figure 3F, **(D)** figure 3G, **(E)** figure 3H, **(F)** figure 3I, **(G)** figure 3J, **(H)** figure 3K, **(I)** figure 3L, **(J)** figure 3M and **(K)** figure EV5B derived by using ImageJ is shown (n=2 independent experiments).

Appendix Figure S4: **(A-C)** Cells transduced with either control or specific indicated siRNA/shRNAs were fixed and immunostained with **(A)** PPM1G, **(B)** α-catenin and **(C)** β-catenin antibodies and imaged by using a confocal microscope. Scale bars, 10 μm.

Appendix Figure S1

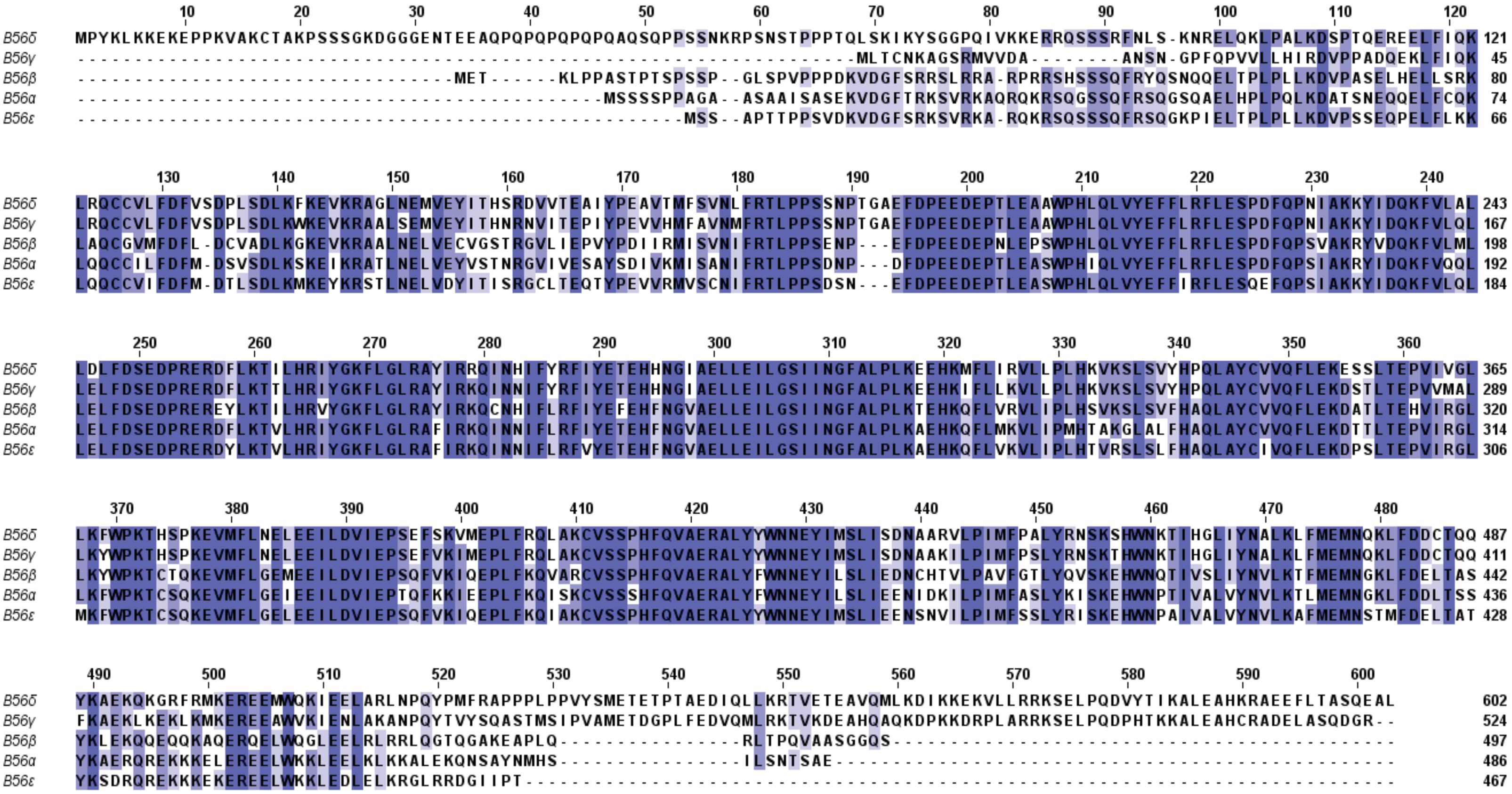

Appendix Figure S2

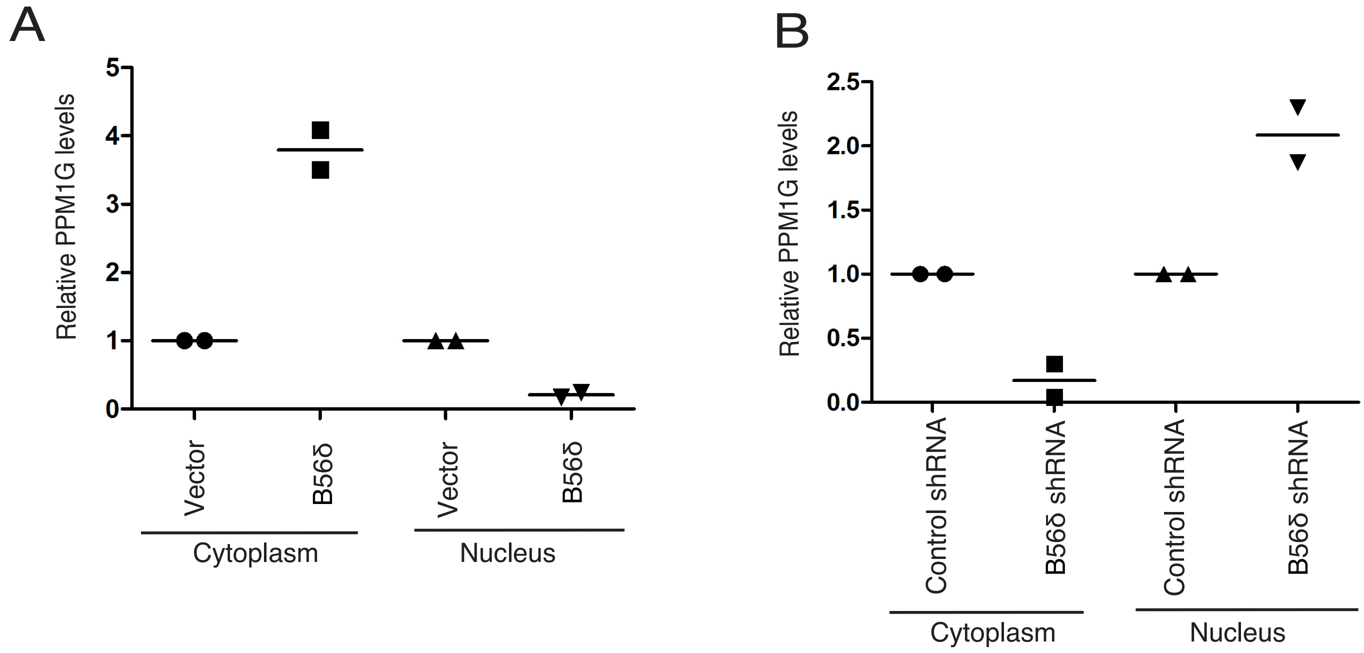

Appendix Figure S3

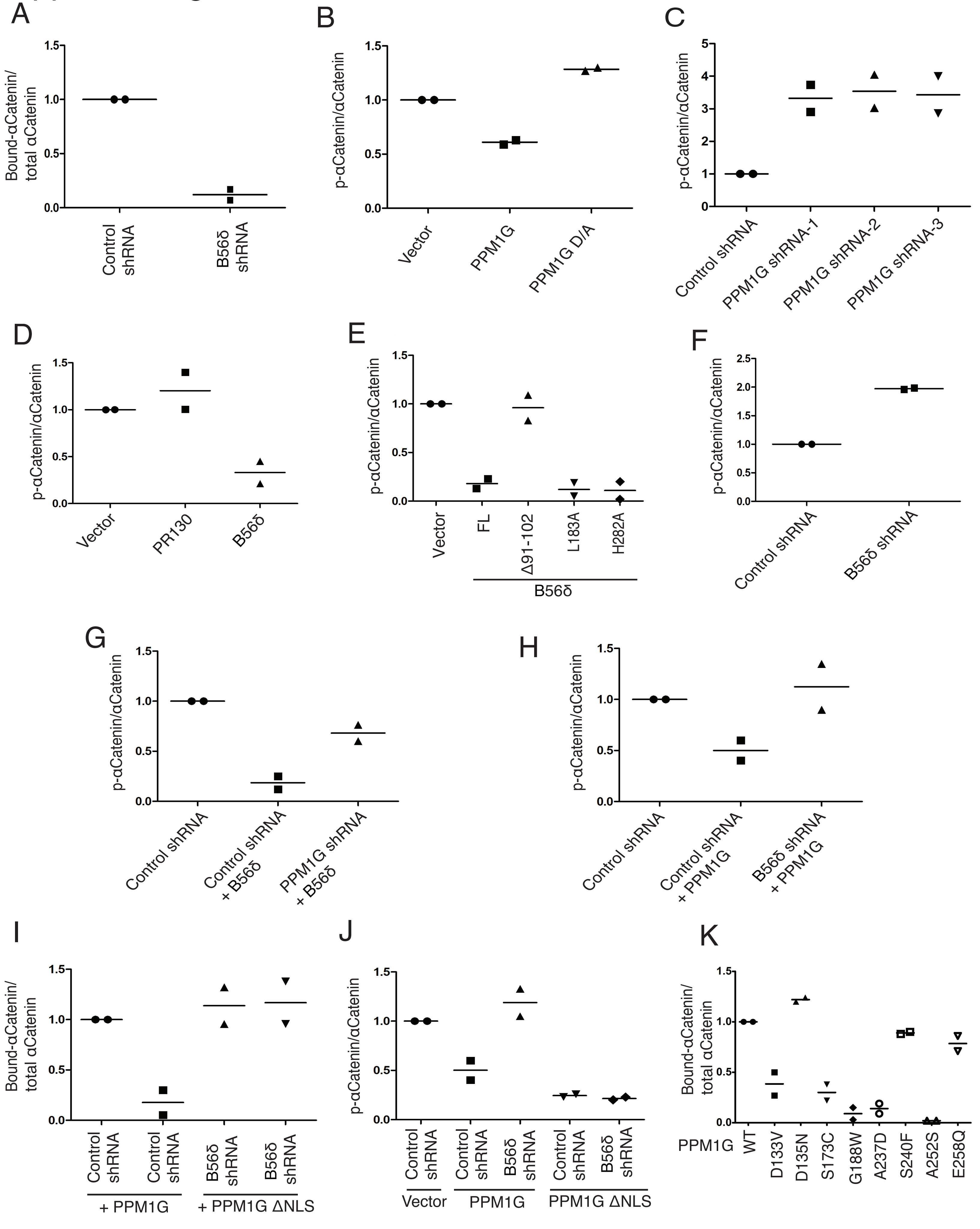

Appendix Figure S4

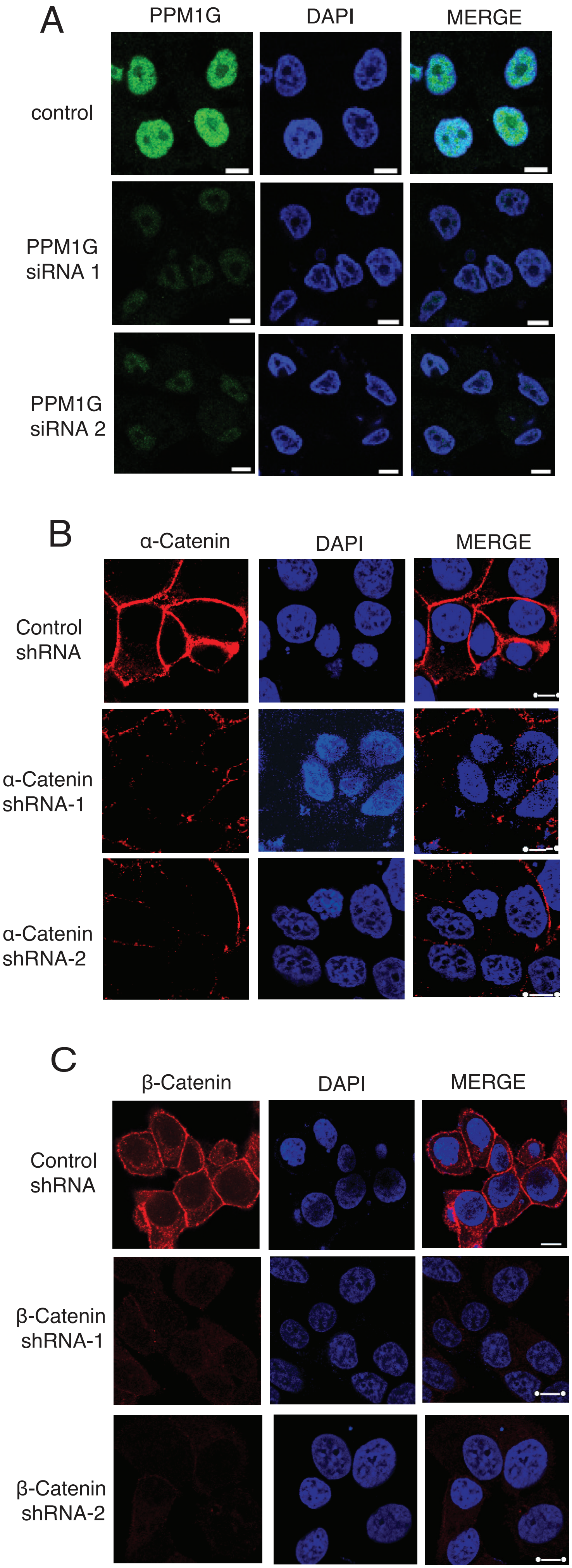

Supplement: Supplementary file 1 — Appendix [file EMBR-20-e46965-s001.pdf]
