# Supplementary material for: PPM1G forms a PPP‐type phosphatase holoenzyme with B56δ that maintains adherens junction integrity
Source: EMBO Rep. 2019 Aug 21;20(10):e46965. doi: 10.15252/embr.201846965 (PMC6776900; doi:10.15252/embr.201846965)
Supplement: Supplementary file 2 — Expanded View Figures PDF [file EMBR-20-e46965-s002.pdf]

Expanded View Figures

Figure EV1. Characterization of PPM1G-B56δ interaction.

A Glutathione Sepharose beads bound with bacterially expressed recombinant GST or GST-B56δ proteins were incubated with bacterially purified recombinant MBP-PPM1G, and interaction of B56δ with PPM1G was detected by immunoblotting with MBP antibody. Recombinant protein expression is shown by Coomassie staining.
B Cells expressing SFB vector, SFB-B56β, SFB-B56ε, and SFB-PPM1G were lysed and pulled down with streptavidin beads. Interaction of B56δ with these proteins was detected by immunoblotting with specific antibody.
C Schematic representation of B56δ full length (FL), and various deletion domains.
D B56δ FL and various deletion domains were expressed and pulled down using streptavidin beads. Interaction of PPM1G with these proteins was detected by immunoblotting using PPM1G antibody.
E Interaction of PPM1G with SFB-tagged B56δ full length (FL) and its N-terminal deletion mutants was detected using pull down with streptavidin beads and immunoblotting by PPM1G antibody.
F Interaction of Myc-tagged PPP2R1A with SFB-tagged B56δ wild type (WT) or its various point mutants was detected using pull down with streptavidin beads and immunoblotting by myc antibody.
G Glutathione Sepharose beads bound with GST, GST-B56δ, or GST-B56δ Δ91–102 proteins were incubated with bacterially purified recombinant MBP-PPM1G, and interaction of PPM1G was detected by immunoblotting with MBP antibody.
H Bacterially purified recombinant GST, GST-B56δ, and GST-B56δ Δ91–102 immobilized on glutathione Sepharose beads were incubated with bacterially purified recombinant MBP-PPP2R1A, and interaction of PPP2R1A was detected by immunoblotting with MBP antibody.
I Binding affinities of wild type (WT), L183A, and H282A mutants of B56δ with PPP2R1A and PPM1G were determined by bio-layer interferometry. 6xHis-tagged PPM1G and PPP2R1A were immobilized on Ni-NTA biosensors and incubated with the GST-B56δ wild type (WT) or L183A and H282A mutants at indicated concentrations. Curves represent experimental trace obtained from the BLI experiments (n = 3 independent experiments).
J GST-B56δ bound on glutathione Sepharose beads were first incubated with MBP-PPP2R1A. The B56δ-PPP2R1A complex was treated with increasing concentrations of His-PPM1G, and its effect on interaction of B56δ-PPP2R1A interaction was assessed by immunoblotting with MBP antibody after pull down with GST beads.

Source data are available online for this figure.

© 2019 The Authors

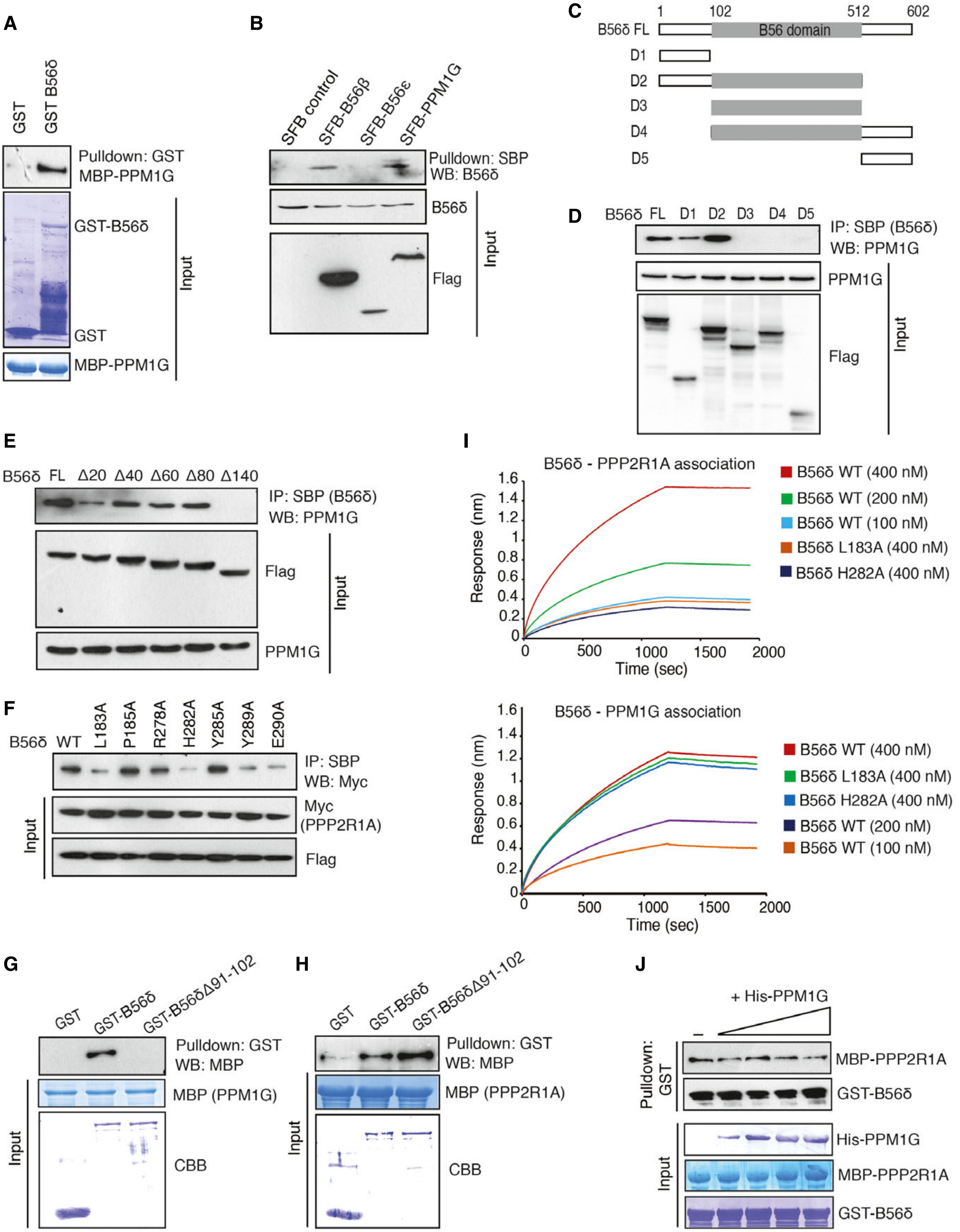
A
GST
GST B56δ
Pulldown: GST
MBP-PPM1G
GST-B56δ
GST
MBP-PPM1G
Input
B
SFB control
SFB-B56β
SFB-B56ε
SFB-PPM1G
Pulldown: SBP
WB: B56δ
B56δ
Flag
Input
C
1
102
512
602
B56δ FL
B56 domain
D1
D2
D3
D4
D5
D
B56δ FL D1 D2 D3 D4 D5
IP: SBP (B56δ)
WB: PPM1G
PPM1G
Flag
Input
E
B56δ FL Δ20 Δ40 Δ60 Δ80 Δ140
IP: SBP (B56δ)
WB: PPM1G
Flag
PPM1G
Input
F
B56δ WT L183A P185A R278A H282A Y285A Y289A E290A
IP: SBP
WB: Myc
Myc
(PPP2R1A)
Flag
Input
I
B56δ - PPP2R1A association
Response (nm)
Time (sec)
B56δ WT (400 nM)
B56δ WT (200 nM)
B56δ WT (100 nM)
B56δ L183A (400 nM)
B56δ H282A (400 nM)
B56δ - PPM1G association
B56δ WT (400 nM)
B56δ L183A (400 nM)
B56δ H282A (400 nM)
B56δ WT (200 nM)
B56δ WT (100 nM)
G
GST
GST-B56δ
GST-B56δΔ91-102
Pulldown: GST
WB: MBP
MBP (PPM1G)
CBB
Input
H
GST
GST-B56δ
GST-B56δΔ91-102
Pulldown: GST
WB: MBP
MBP (PPP2R1A)
CBB
Input
J
+ His-PPM1G
–
Pulldown: GST
MBP-PPP2R1A
GST-B56δ
His-PPM1G
MBP-PPP2R1A
GST-B56δ
Input

Figure EV1.

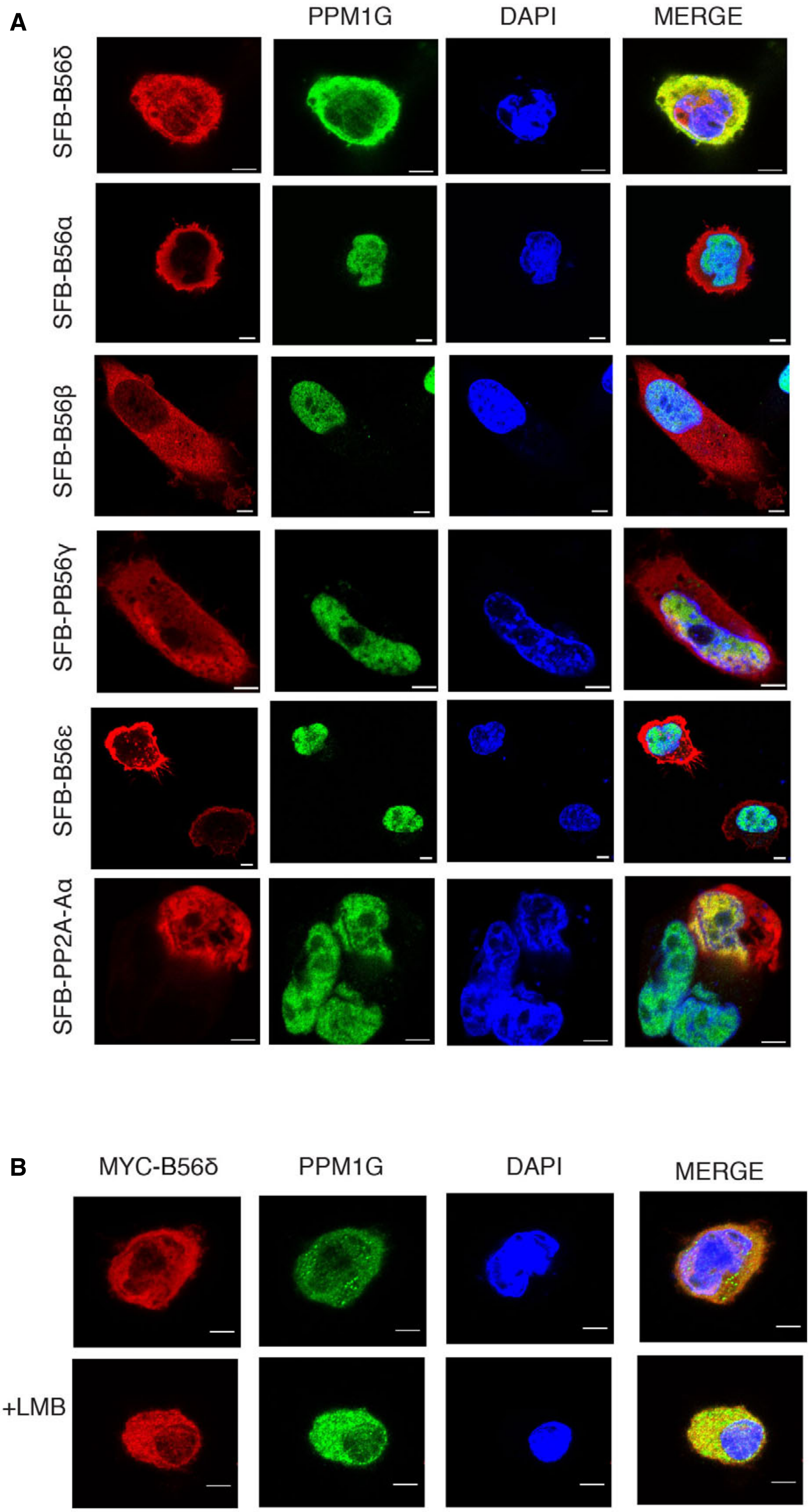

Figure EV2. Effect of B56δ on PPM1G localization.

A SFB-B56α, SFB-B56β, SFB-B56γ, SFB-B56ε, and SFB-PP2A-Aα were expressed in MDA-MB-231 cells, and PPM1G localization was determined by confocal imaging after staining with PPM1G antibodies. Scale bar: 5 μm.

B Myc-B56δ expressing MDA-MB-231 cells were treated with Leptomycin B (LMB) for 3 h, and then, cells were fixed and imaged using MYC and PPM1G specific antibodies. Scale bar: 5 μm.

 © 2019 The Authors

Figure EV3. Characterization of PPM1G-α-catenin interaction.

A List of HCIPs (high confidence interacting proteins) found in purification of SFB-PPM1G using tandem affinity purification and mass spectrometric identification.
B SFB-α-catenin and Myc-PPM1G were expressed in 293T cells. Interaction of PPM1G with α-catenin was detected by Western blotting with Myc antibody after immunoprecipitation with either IgG or Flag antibody.
C Nuclear and cytoplasmic fractions were collected from cells expressing SFB-α-catenin. Interaction of α-catenin with PPM1G and β-catenin was detected by immunoprecipitation with Flag followed by blotting with PPM1G and β-catenin antibody, respectively.
D Schematic representation of PPM1G full length (FL), along with its various deletion mutants (PD: phosphatase domain, AD: acidic domain).
E 293T cells were co-transfected with the indicated SFB-tagged PPM1G constructs along with Myc-tagged α-catenin, and their interaction was determined by pull down using streptavidin beads followed by immunoblotting with Myc antibody.
F SFB-tagged PPM1G wild type (WT) or PPM1G ΔNLS mutant were expressed in cells, and their localization was determined by immunostaining with Flag antibody. DAPI was used to counterstain nucleus.

© 2019 The Authors

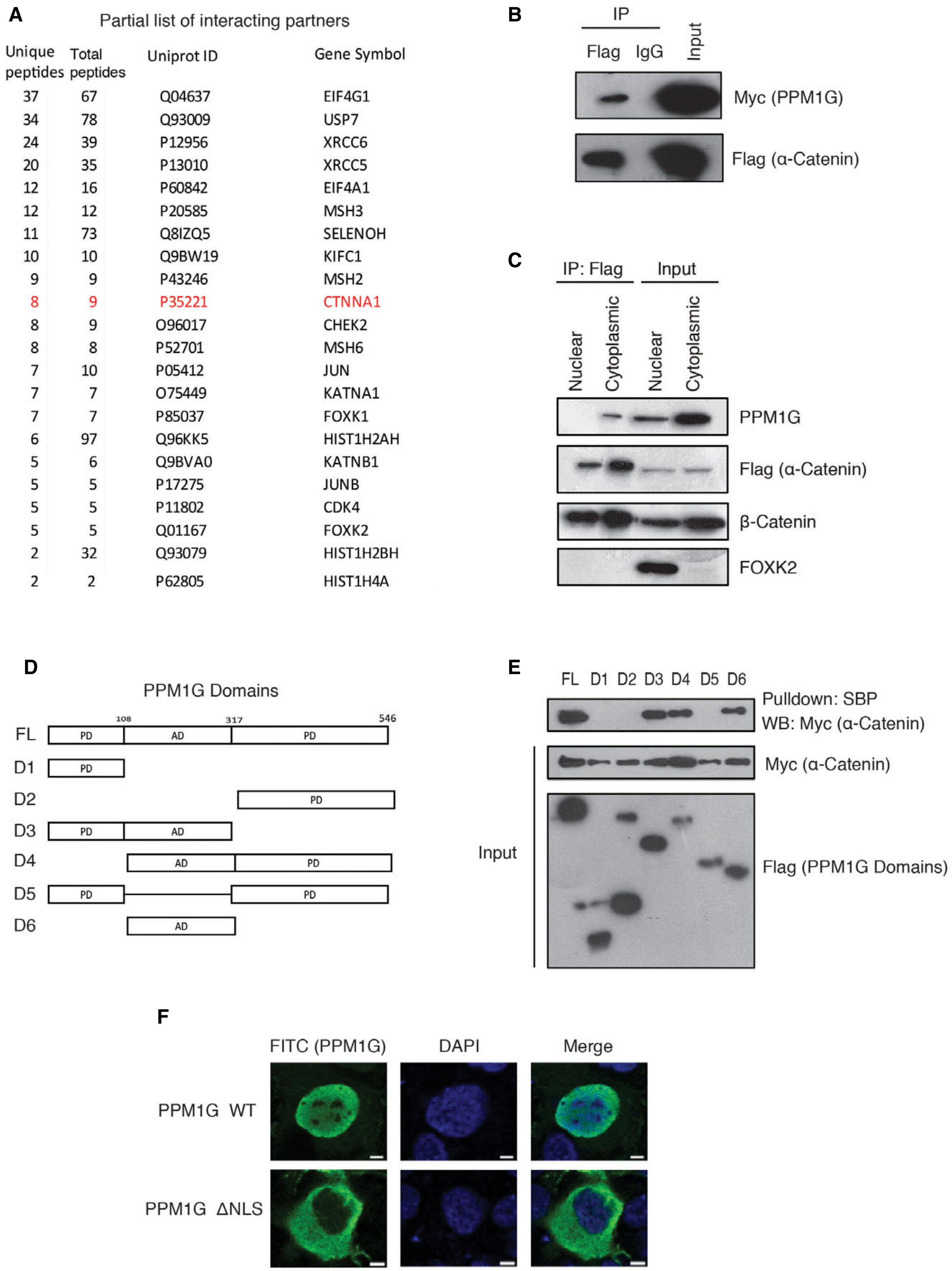

Unique peptides	Total peptides	Uniprot ID	Gene Symbol
37	67	Q04637	EIF4G1
34	78	Q93009	USP7
24	39	P12956	XRCC6
20	35	P13010	XRCC5
12	16	P60842	EIF4A1
12	12	P20585	MSH3
11	73	Q8IZQ5	SELENOH
10	10	Q9BW19	KIFC1
9	9	P43246	MSH2
8	9	P35221	CTNNA1
8	9	O96017	CHEK2
8	8	P52701	MSH6
7	10	P05412	JUN
7	7	O75449	KATNA1
7	7	P85037	FOXK1
6	97	Q96KK5	HIST1H2AH
5	6	Q9BVA0	KATNB1
5	5	P17275	JUNB
5	5	P11802	CDK4
5	5	Q01167	FOXK2
2	32	Q93079	HIST1H2BH
2	2	P62805	HIST1H4A

Figure EV3.

 © 2019 The Authors

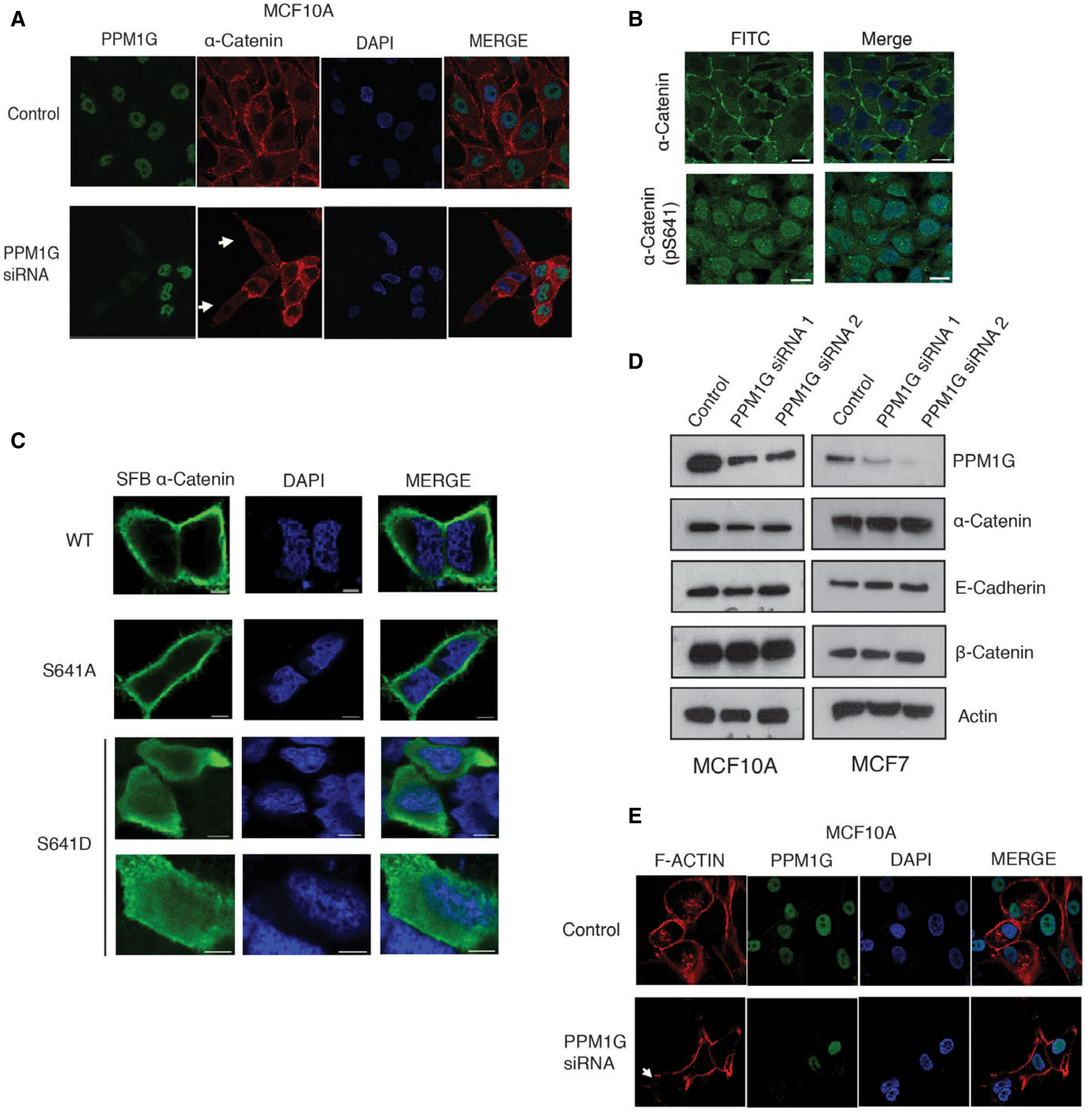

Figure EV4. Role of PPM1G-B56δ on α-catenin localization at adherens junctions.

A MCF10A cells were transfected with either control siRNA or PPM1G siRNA, and the localization of α-catenin was determined by using immunofluorescence with a specific antibody. Arrows indicate α-catenin staining in knock down cells.

B Localization of α-catenin and pS641 α-catenin was detected by immunofluorescence after staining the cells with specific antibodies. Scale bars, 20 μm.

C SFB-tagged α-catenin, S641A, and S641D mutants were expressed, and their localization was detected by staining with Flag antibody. Scale bars, 5 μm.

D Either control or two different PPM1G siRNAs were transfected into MCF10A and MCF7 breast epithelial cells. Levels of indicated proteins were determined by Western blotting with specific antibodies.

E F-Actin in control and PPM1G-depleted cells was stained using rhodamine-labeled phalloidin, and the actin cytoskeleton was visualized by using confocal microscope. Arrows indicate F-actin staining in knock down cells.

Figure EV5. Effect of PPM1G-B56δ on cell migration.

A Transwell migration assays were performed with MCF10A cells expressing either control siRNA or two different PPM1G siRNAs. Quantification data for migrated cells per field (derived from average from 3 different fields) from three independent experiments are shown. Error bars indicate standard deviation ($n = 3$), ***$P < 0.001$ (one-way ANOVA, post hoc test: Bonferroni's multiple comparison test).

B Various SFB-tagged acidic domain mutants of PPM1G derived from COSMIC database were expressed in 293T cells, and their interaction with α-catenin was detected by immunoblotting with α-catenin antibody after pull down using streptavidin beads.

C Phosphatase activity of bacterially purified recombinant PPM1G wild type (WT) or various point mutants was measured by using pNPP as a substrate. Amount of released phosphate from pNPP was assayed colorimetrically. Error bars indicate standard deviation ($n = 3$ independent experiments), ***$P < 0.001$ (one-way ANOVA, post hoc test: Bonferroni's multiple comparison test).

D, E Various SFB-tagged acidic domain mutants of PPM1G were expressed in MDA-MB-231 cells. Representative images from transwell migration assay (D) are shown, and (E) quantified data for migrated cells per field from three independent experiments were plotted. Error bars indicate standard deviation ($n = 3$), ***$P < 0.001$ (one-way ANOVA, post hoc test: Bonferroni's multiple comparison test).

F Data from the migration assays performed with MCF10A cells expressing either control shRNA or two different B56δ shRNAs were plotted. Error bars indicate standard deviation ($n = 3$ independent experiments), ***$P < 0.001$ (one-way ANOVA, post hoc test: Bonferroni's multiple comparison test).

G MDA-MB-231 cells expressing either control shRNA, PPM1G shRNA, or B56δ shRNA were plated in equal numbers, and rate of cell proliferation for the indicated days after plating, derived by cell counting, was plotted. Error bars indicate standard deviation ($n = 3$ independent experiments); n.s (one-way ANOVA, post hoc test: Bonferroni's multiple comparison test).

H SFB-B56δ was expressed in control shRNA and PPM1G shRNA-transduced MCF7 cells, and expression of different proteins was determined by immunoblotting with the indicated antibodies.

I SFB-PPM1G was expressed in control shRNA and B56δ shRNA-transduced MCF7 cells, and expression of different proteins was determined by immunoblotting with the indicated antibodies.

J Control shRNA and B56δ shRNA-transduced MCF7 cells were transfected with MYC-PPM1G and MYC-PPM1G ΔNLS, and expression of different proteins was determined by immunoblotting with the indicated antibodies.

K SFB-tagged α-catenin S641A and S641D mutants were expressed in PPM1G shRNA and B56δ shRNA-transduced MCF7 cells, and expression of different proteins was determined by immunoblotting with the indicated antibodies.

Source data are available online for this figure.

 © 2019 The Authors

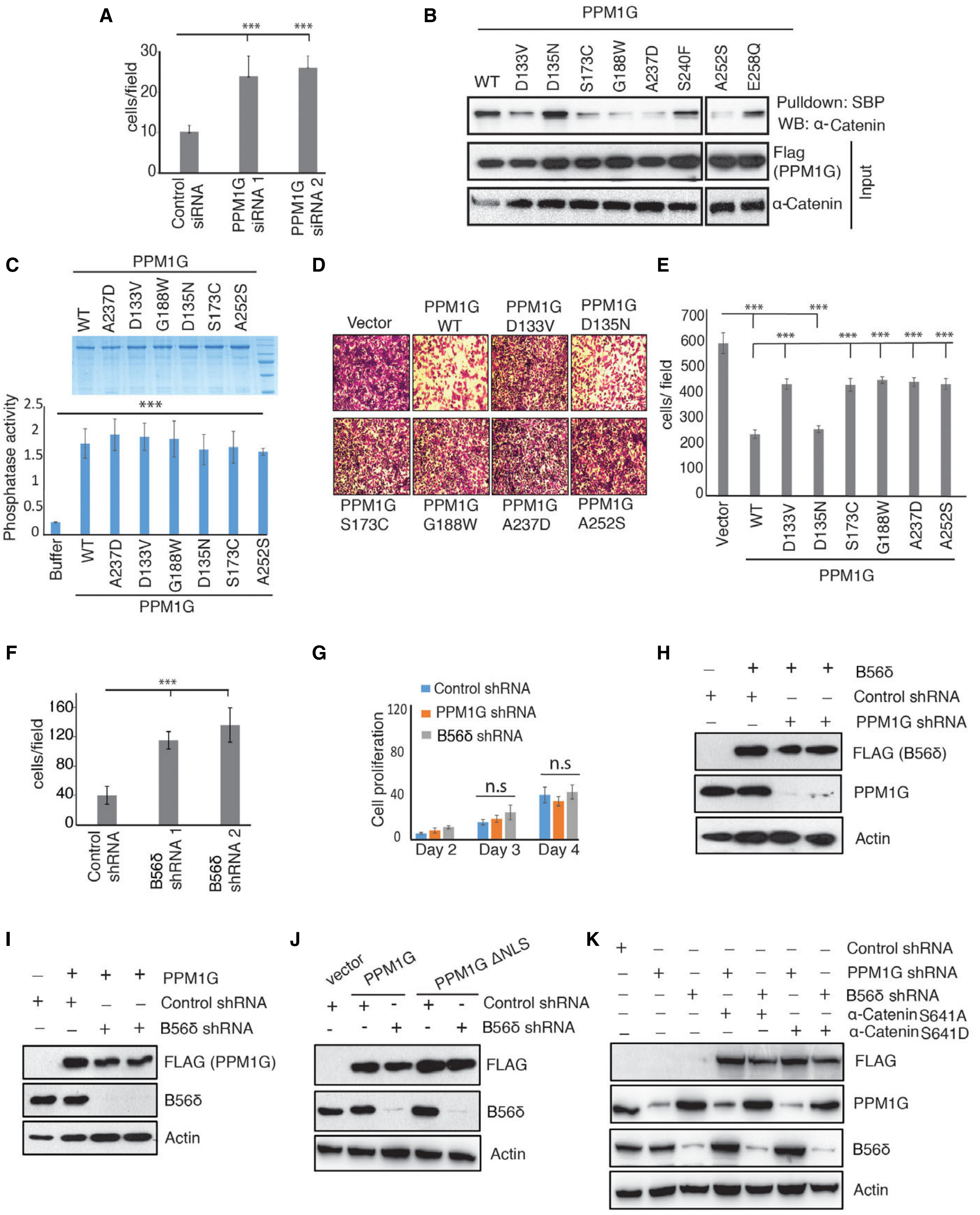
A
cells/field
Control siRNA
PPM1G siRNA 1
PPM1G siRNA 2
B
PPM1G
WT D133V D135N S173C G188W A237D S240F A252S E258Q
Pulldown: SBP
WB: α-Catenin
Flag (PPM1G)
α-Catenin
Input
C
PPM1G
WT A237D D133V G188W D135N S173C A252S
Phosphatase activity
Buffer
D
Vector
PPM1G WT
PPM1G D133V
PPM1G D135N
PPM1G S173C
PPM1G G188W
PPM1G A237D
PPM1G A252S
E
cells/ field
Vector WT D133V D135N S173C G188W A237D A252S
PPM1G
F
cells/field
Control shRNA
B56δ shRNA 1
B56δ shRNA 2
G
Control shRNA
PPM1G shRNA
B56δ shRNA
Cell proliferation
n.s
Day 2 Day 3 Day 4
H
B56δ
Control shRNA
PPM1G shRNA
FLAG (B56δ)
PPM1G
Actin
I
PPM1G
Control shRNA
B56δ shRNA
FLAG (PPM1G)
B56δ
Actin
J
vector
PPM1G
PPM1G ΔNLS
Control shRNA
B56δ shRNA
FLAG
B56δ
Actin
K
Control shRNA
PPM1G shRNA
B56δ shRNA
α-Catenin S641A
α-Catenin S641D
FLAG
PPM1G
B56δ
Actin

Figure EV5.
