# Supplementary figures and images for: PPM1G forms a PPP‐type phosphatase holoenzyme with B56δ that maintains adherens junction integrity
Source: EMBO Rep. 2019 Aug 21;20(10):e46965. doi: 10.15252/embr.201846965 (PMC6776900; doi:10.15252/embr.201846965)

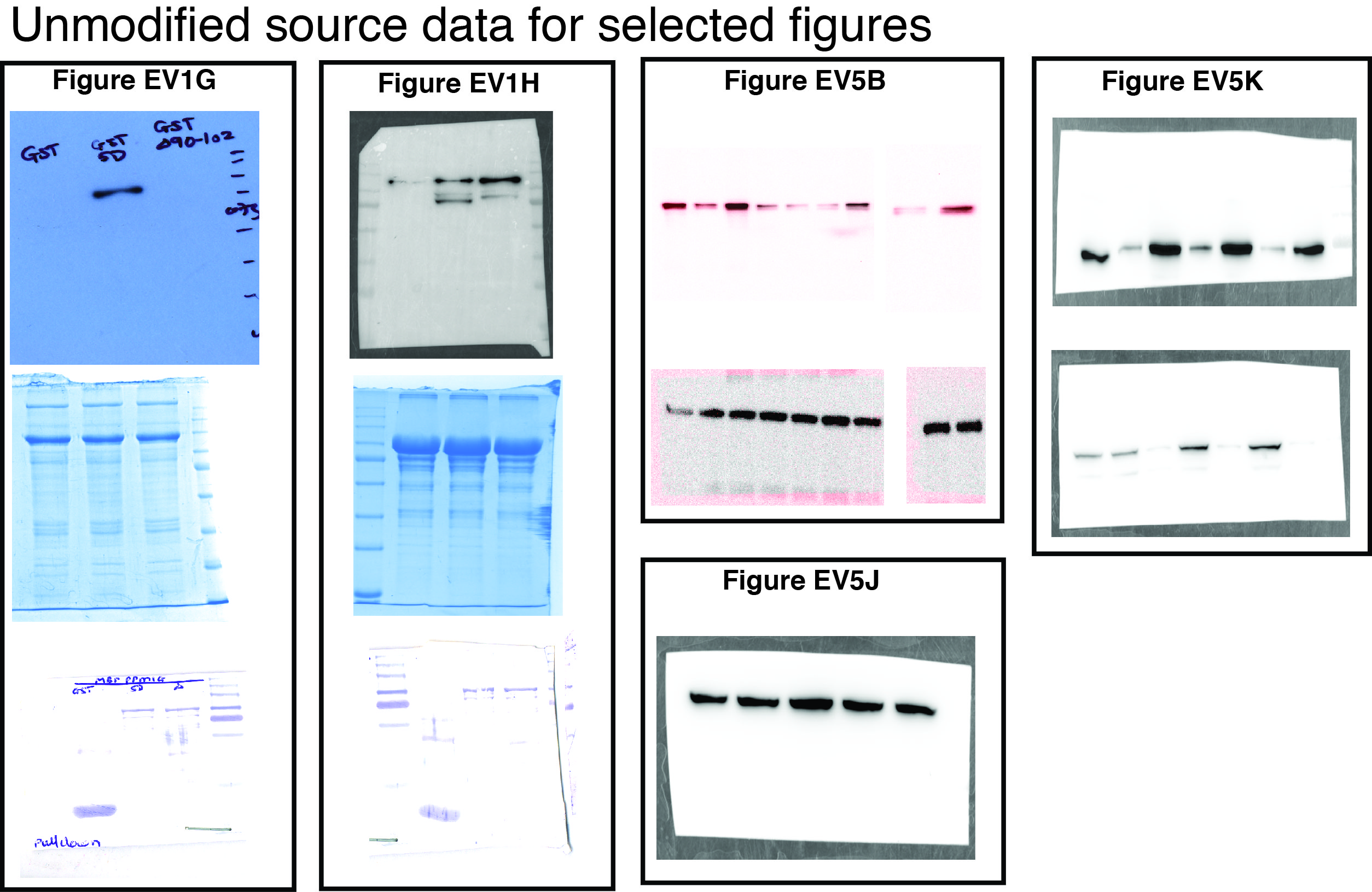

Supplement: Supplementary file 6 — Source Data for Expanded View [file EMBR-20-e46965-s006.jpg]
